# Correspondence

**Published:** 1890-03

**Authors:** C. D. Roy

**Affiliations:** Charity Hospital; New York


NEW YORK.

The influenza, which has seemingly laid violent hands upon the dwellers in Gotham, has not only opened a field of incessant labor to the city practitioners, but our hospital itself has been crowded with patients of pneumonitis, the resultant, as it were, of the prevailing epidemic. There have been several causes entering as factors in the production of the increased bronchial troubles which have occurred in the city this winter. One of the chief has been the unseasonableness of the weather which has been experienced, the moderately cold, damp atmosphere being so suitable for the prevailing acute troubles. Another of very little lesser importance has been the laying of the sub-ways in the city, thus necessitating the tearing up of the streets and the laying bare of soil impregnated with the city's filth. These, with the uncleanliness of the streets, have made the people more susceptible to the complaints which are prevalent at this season of the year, and which have flourished upon the soil thus made vulnerable. The coldness of the weather which occurs in New York is made much more penetrable from the fact that it is very seldom unassociated with a damp atmosphere. We who live upon the Island become accustomed to the dampness from the proximity of water on all sides. Blackwell's Island, to those who have never visited the spot and know nothing of its surroundings, has no other significance than the fact of its being the location of the city penitentiary. There are upon it, however, other important city institutions, and to those who may have the opportunity, a trip to the island will prove most pleasant and profitable. A description of the institutions, however, would be out of place in a medical journal, but I shall take the liberty of speaking a few words about Charity Hospital, which may prove of some interest to the readers of the Record.

The main building of the hospital is a granite structure of four stories in height, built in the shape of the letter H. In this building there are twenty-six wards, thirteen for each sex. Besides this, the resident staff has charge of the New York Maternity and Epileptic Hospitals, which are distinct buildings, situated in close proximity to the main structure. The inmates of the penitentiary, numbering on an average of eight hundred, are also under the supervision of the same medical charge. The hospital proper contains eleven hundred beds, and there is treated during the year an average of eight thousand patients. All classes of diseases are seen throughout the year, although it is especially known for its venereal service, this being the largest service of its kind on this side of the ocean. Here a person has the opportunity for studying all classes of venereal troubles, and every syphilide can be seen in the various wards as is shown in the cases represented in the "Atlas of Venereal Diseases" by Dr. R. W. Taylor.

In some later letter I shall give the methods in the management of the Maternity Hospital, which shall be practical in their character, and points which are often overlooked and which are so necessary for the successful practice of obstetrics.

Soon after my entrance into the hospital, there occurred upon the medical service with which I was connected, a case of much interest and one of very unique character. A Bohemian girl, aged 19, was admitted into the hospital about the 15th of August, and transferred to a medical ward under the diagnosis of simple ulcer of the stomach. From the character of her symptoms and history, the latter of which being very meagre, the visiting and house physicians confirmed the existing diagnosis. When I entered upon the service, on the 1st of October, the following symptoms were found to be present: Subjectively, the patient complained of pain in the epigastric region, which was especially intensified on taking food. There was occasionally nausea and vomiting, accompanied frequently with attacks of hæmatemesis. Objectively, there was pain on pressure below the ensiform cartilage, and the patient continuously wore a painful and anxious expression. The patient was placed upon fluid diet for three weeks, with such marked improvement that at the end of that time she desired to go from the hospital. So improved was she that without the knowledge of the nurse, she partook of some solid food which had been brought her by a friend. This last act caused a return of her symptoms, with increased severity. On October 29th I was called up twice during the night to check the vomiting of blood. This I accomplished by the administration of a drachm of the fluid extract of ergot, allowing her to swallow small particles of ice and giving her hypodermatically ten minims of Magendies solution, which is about equal to 1-3 gr. morphine, so as to relieve her pain. The next day she had three attacks of hæmatemesis, and on the second day she had two more. The last one was the crisis, for with the vomited materials there was also ejected a small sized dressing pin. From that moment her pain was relieved so that she gradually improved, and on the fifth day she was discharged from the hospital. Two days before her departure, she was allowed to partake of all solid food with no attending inconveniences. The patient could not speak English, so that her personal history was necessarily incomplete.

I mention this case simply to show a new factor which enters into the differential diagnosis of gastric ulcers.

Having had occasion in the wards to try for myself the comparatively recent treatment of gonorrhœa by the irrigation of the urethra, with a bi chloride solution, it may be of some interest to those who have not yet tried the method, to know some of its advantages and disadvantages. The former are few, while the latter are many. The strength of the solution used is 1-40000, or a little less than one-fifth of grain to the pint of water. This is the strength of the solution used in the hospital, although Keyes, in his most excellent book, advises a solution of 1-20000.

The irrigation is carried out as follows: A reservoir containing the solution is placed at a small height above the patient's head, who is made to sit upon the edge of a chair so that there shall be no wetting of the chair or its occupant. To the reservoir is attached a rubber tube, at the further end of which is fastened the apparatus known as Keifer's Irrigator, which is simply two hard rubber tubes joined to a common trunk, which has two distinct openings for the two limbs. Through one of these tubes the solution is allowed to flow into the urethra and out of the other opening.

This is the method of anterior irrigation, as opposed to the deep method, which is seldom resorted to here. The irrigation is applied three times a day until the discharge begins to decrease, when the intervals are somewhat extended.

This plan of treatment is very readily applicable in a hospital where the necessary apparatus is constantly kept and alle th details can be easily carried out, but for private practice it has serious drawbacks. In the first place, and the chief, it does not produce the desired effect, this being in accordance with my observations, and that of others who have seen it tried constantly for a year. Dr. Keyes says that in simple urethretis it will often have the desired effect, but our experience here teaches us that it must be so slight as to hardly need interference. In all cases where the treatment proved of no avail we examined thoroughly to see that there was no lurking strictural to cause the failure of the remedy. The second disadvantage is that it is not suitable for application in private practice, for either the patient must come to your office three times a day or he must fix up his own apparatus and then run the risk of not carrying out its application in a thorough manner. Hence I do not think it is a treatment to be recommended when you wish to cure your case in question.

Another method which I have had some opportunity in testing, and one which I am sorry I cannot recommend, is that which consists in packing the urethra with powder boracic acid and allowing it to be kept so except when washed out by urination. A special packing instrument has been devised for this mode of procedure, but it is unnecessary for me to go into a detailed description. The method was not successful in the cases upon where it was tried, and in two cases slight urethral strictures were occasioned. The method, however, from which we get the best results is the combined internal and mild local treatment.

C. D. ROY, M. D.
Charity Hospital.

